# Lambda Theta Reflectometry: A New Technique for Measuring Optical Film Thickness in Planar Protein Arrays

**DOI:** 10.1021/acssensors.5c01108

**Published:** 2025-07-02

**Authors:** Alanna M. Klose, Joseph D. Katz, Robert Boni, David Nelson, Brian Hassard, Benjamin L. Miller

**Affiliations:** † Department of Dermatology, University of Rochester, Rochester, New York 14627, United States; ‡ Materials Science Program, University of Rochester, Rochester, New York 14627, United States; § Laboratory for Laser Energetics, University of Rochester, Rochester, New York 14627, United States; ∥ Department of Biomedical Engineering, University of Rochester, Rochester, New York 14627, United States; ⊥ Institute of Optics, 6927 University of Rochester, Rochester, New York 14627, United States

**Keywords:** reflectometry, immunoassay, optical thickness measurement, thin film, spectroscopy

## Abstract

Quantitative protein measurements provide valuable information about biological pathways, immune system functionality, and the mechanisms of disease. The most accurate methods for detecting proteins are label-free and preserve native protein-binding interactions. Label-free biomolecular interaction analysis includes reflectometry, a group of techniques that detect proteins by measuring the reflectance properties of a thin film on a substrate. Most of these techniques are limited in some way by instrument complexity, sensitivity, or consumable manufacturing requirements. To address these issues, we introduce Lambda Theta Reflectometry (LTR), a new reflectometric technique that measures changes in film thickness by determining the point of null reflectivity as a function of wavelength (lambda) and angle of incidence (theta). The substrate is simultaneously illuminated with a range of angles and wavelengths, and reflected light is resolved both angularly and spectrally. Our prototype LTR reflectometer can measure SiO_2_ layer thickness with milli-Ångström precision. LTR measurements of Si/SiO_2_ oxide films are in excellent agreement with spectroscopic ellipsometry for film thicknesses ranging from 1390 to 1465 Å. This technique enables label-free biosensing measurements across a range of biological analyte concentrations (0.5 ng/mL to μg/mL) without requiring stringent control over probe deposition thickness or substrate manufacturing.

## Graphical abstract

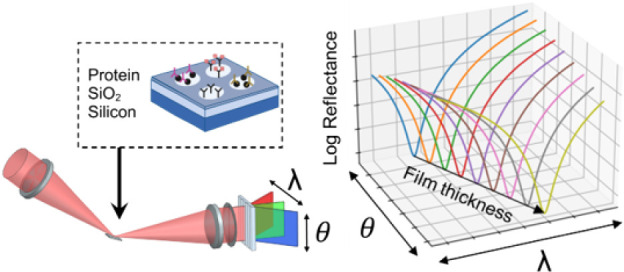

Biosensors detect and quantify proteins, nucleic acids, and small molecules and are essential tools for advancing research and guiding medical treatment.^1^ Label-free biosensors directly measure the interaction of an analyte with a transducer.^2^ The field of label-free optical biosensors^3^ includes surface plasmon resonance,^4^ Raman spectroscopy,^5^ integrated photonic devices,^6^ and reflectometry.^7^ Reflectometry is a group of techniques that measure the optical thickness of a functionalized thin film by illuminating the substrate and evaluating the properties of the reflected light. As light encounters a thin-film layer stack, reflections at the layer boundaries interfere with each other, encoding information about layer properties into the reflected light. Biological binding events, such as a protein binding to its receptor or an antibody binding to its antigen, cause Ångström-level increases in film thickness as bound and unbound probe sites are averaged by the optical measurement.

Reflectometric biosensors detect binding events by observing the characteristics of the reflected light, such as the polarization state, spectral interference patterns, or reflectivity at a fixed angle or wavelength. Ellipsometry^8^ measures change in the polarization of reflected light. Oblique-Incidence Reflectivity Difference (OI-RD) microscopy^9^ measures the reflectivity difference between p-polarized and s-polarized light. Reflectometric Interference Spectroscopy (RIfS)^10^ and Biolayer Interferometry (BLI)^11^ illuminate a thin-film stack at normal or near-normal incidence and evaluate changes in the interference pattern of reflected light as a function of wavelength. Other techniques measure a change in the reflected light intensity at a single or a few discrete wavelengths at a fixed angle (Spectral Reflectance Imaging Biosensor (SRIB/IRIS),^12^ 1λ Reflectometry,^13^ and Arrayed Imaging Reflectometry (AIR)).^14^ AIR employs a single wavelength and angle of incidence (AOI) to measure analyte binding via increased reflectance from an antireflective condition. AIR is unique among reflectometry techniques because it leverages the steep drop in reflectivity under absolute antireflective conditions.

An absolute antireflective condition occurs when light waves reflecting off the boundaries of the thin film meet with equal amplitude and opposite phase, such that the sum of reflected waves equals zero. An example using a commonly employed material stack consisting of silicon, SiO_2_, and air is shown in Figure 1a. When waves of equal amplitude meet, total destructive interference occurs when the optical path length of light traveling through layer *n*_SiO2_ is exactly a half-integer multiple of the wavelength of the incident light. With the appropriate selection of illumination polarization, wavelength, AOI, and layer thickness, the condition of zero reflectance can theoretically be achieved (Figure 1b). When the optical thickness (*n*_SiO2_ *d*) changes, the antireflective condition exists at a different combination of illumination wavelength and AOI. In the context of biosensing, the silicon dioxide is functionalized with probes that have specific affinity to the analyte of interest. Upon binding of the analyte, the average thickness of the layer increases according to the fractional coverage of binding sites.^15^

**Figure 1:**
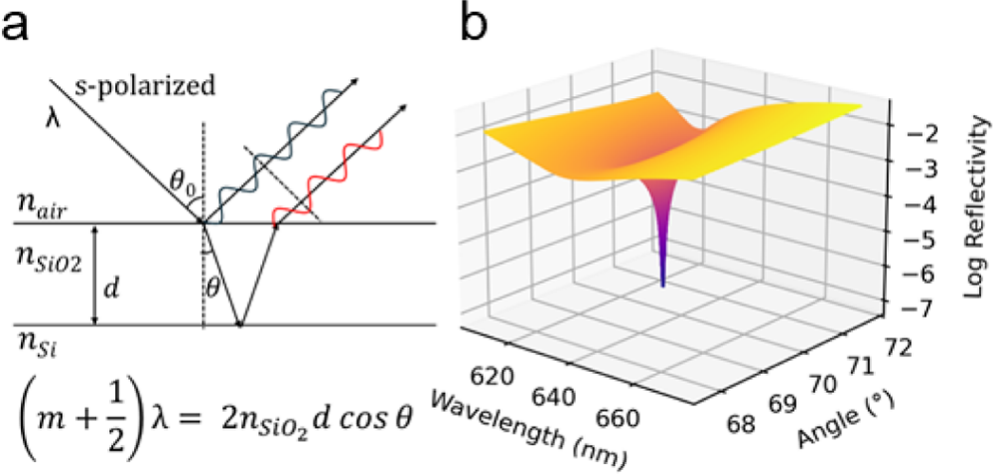
(a) The null reflectivity condition is a function of the incident light angle, wavelength, and film thickness. While the film is shown in this diagram as silicon dioxide for simplicity, this phenomenon includes biofunctionalized SiO_2_. (b) A modeled null condition with a steep drop in reflectivity exists at a specific combination of wavelength and AOI for a thin film with a given optical thickness.

AIR is most accurate only if the thicknesses of the starting SiO_2_ film and adhesion chemistry (typically a silane carrying an electrophilic moiety enabling covalent attachment of probes) are controlled precisely, and probes are deposited at the optimal thickness required to produce an antireflective condition prior to analyte capture.^16^ The relationship between the measured AIR signal and the substrate thickness is established empirically via cross-calibration of unfunctionalized Si/SiO_2_ substrates to ellipsometry and is represented by a parabolic fit. As such, layer thickness is not uniquely determined by the data for conditions that deviate from the reflectance null. In practice, for higher-plex biosensing arrays, when many different probes are deposited in different positions on a monolithic substrate, the required control over baseline thickness at each site is difficult to achieve. This limitation of AIR motivated the design of a new reflectometric instrument that decouples measurement accuracy from baseline probe thickness while maintaining the steep slope of the antireflective condition, enabling high-resolution measurements.

Lambda Theta Reflectometry (LTR) simultaneously illuminates the substrate surface with a cone of AOIs and a continuous spectrum of wavelengths to ensure that the null reflectance condition is sampled regardless of the initial substrate conditions. By locating the coordinates of the reflectance null in wavelength and angle space, a unique determination of the substrate thickness can be made with milli-Ångström precision. Sampling the reflectance parameters around an absolute null produces a data set that is highly sensitive to illumination conditions as the relative change in reflectance, given by ▽*R*(λ, θ)/*R*, increases asymptotically as the null is approached. This condition can only be realized by isolating the illumination properties: λ, θ, and polarization. The depth of the null is theoretically infinite, offering a feature size that is limited only by the measurement resolution in lambda–theta space. The statistical uncertainty associated with locating the centroid of a function scales with both the width and the gradient of the function.^17^ As a result, the centroid position of a data set that is narrow and steep can be found more accurately than that of a data set that is wide and shallow. In this respect, LTR differs from other reflectometry techniques that do not uniquely isolate the illumination conditions required to produce an absolute null and therefore have limited measurement contrast.

The prototype LTR instrument (Figure 2a) measures the reflectivity of a biofunctionalized Si/SiO_2_ substrate with continuous angular and spectral resolution spanning 68°–74° and 590–670 nm. The fiber optic output of the lamp is collimated, polarized, and imaged on the tilted substrate surface, resulting in an elliptical illumination region defined by the fiber core size and the substrate tilt angle. Using a 50-μm core fiber results in a spot with major and minor axes of 150 and 50 μm, respectively. The surface of the substrate is reimaged onto a field stop, providing a means to further reduce the size and shape of the analysis region. S-polarized light was selected to provide the appropriate reflection amplitudes for a fully destructive interference. The illumination angle and wavelength parameters were selected to measure the changes in film thickness over a 100 Å range from 1390 to 1490 Å.

**Figure 2:**
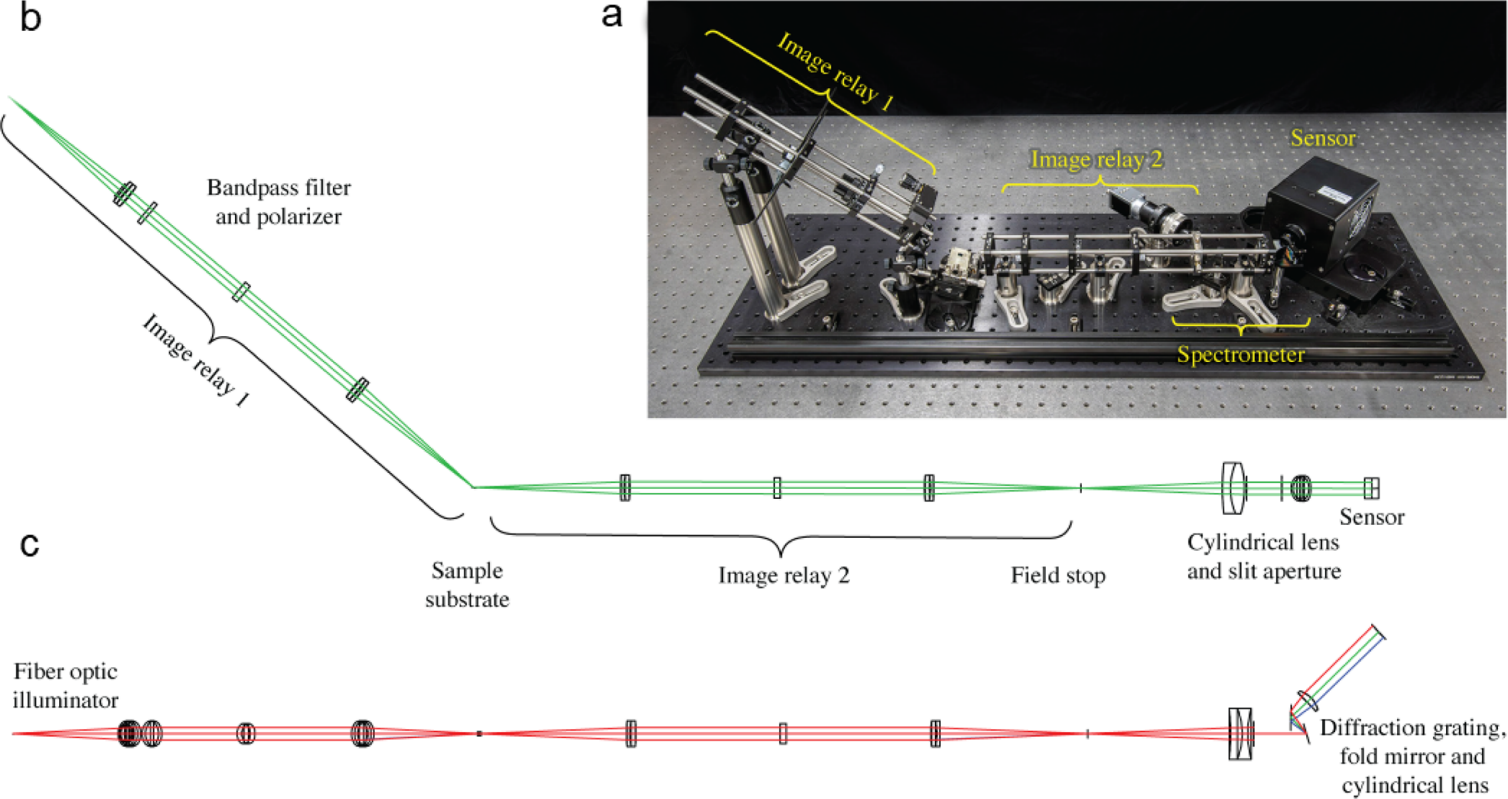
LTR angularly and spectrally resolves the surface reflectance of a sample simultaneously without any moving parts by dispersing the light onto a CCD detector. Two orthogonal projections of the optical layout are shown. (a) A photo of the instrument with a light-tight enclosure removed. (b) A side-on view displays the ray fans that are angularly resolved. The range of angles that are sampled (68–74°) is determined by the sample holder tilt angle and the numerical aperture of the illumination image relay. (c) A top–down view displaying the ray fans that are spectrally resolved. A bandpass filter limits the wavelengths sampled to 590–670 nm. A slit aperture rejects rays that are incident on the sample at compound angles relative to the angular resolution plane. A diffraction grating disperses the remaining rays as a function of the wavelength.

Cylindrical optics, a slit aperture, and a diffraction grating are used to orthogonally disperse the reflected light based on the AOI (Figure 2b) and the illumination wavelength (Figure 2c) producing a two-dimensional image that is captured using a CCD camera. The resulting reflectivity data are related to the average thickness of the illumination region. Moving the stage holding the substrate relative to the fiber core image allows different locations on the substrate surface to be sampled. A secondary alignment camera views the surface to facilitate the alignment of the illumination region to individual probe sites deposited on the substrate.

## Results and Discussion

To demonstrate the functionality of the LTR technique in a simple scenario, we first measured the oxide thickness of a clean Si/SiO_2_ substrate. Each pixel in the experimental LTR image corresponds to a reflectance measurement at a particular wavelength and AOI (Figure 3a). The data reduction process returned film thickness by identifying the conditions of best fit between modeled and measured reflectivities over an entire LTR image. Theoretical reflectance over a range of wavelengths and angles was calculated as a discrete function of film thickness using the characteristic matrix method for multilayered thin films.^18^ The theoretical reflectivity of pure s-polarized light incident on an atomically smooth and flat substrate drops to zero, but finite spectral and angular resolution, scattered light at the detector plane, and thin-film substrate imperfections limit the reflectivity depth of what can practically be observed. The typical minimum measured reflectivity for our Si/SiO_2_ substrates was ∼5 × 10^–4^. Three potential sources were considered that could contribute to the experimentally observed null depth. These were the s:p polarization ratio, substrate thickness variation at a length scale comparable to the illumination wavelengths used, and scattered light. Reducing the modeled polarization s:p contrast ratio from what was expected when using a Glan-Thompson calcite polarizer from 100 000:1 to 50 000:1 was not sufficient to account for the full difference in minimum reflectivity.

**Figure 3:**
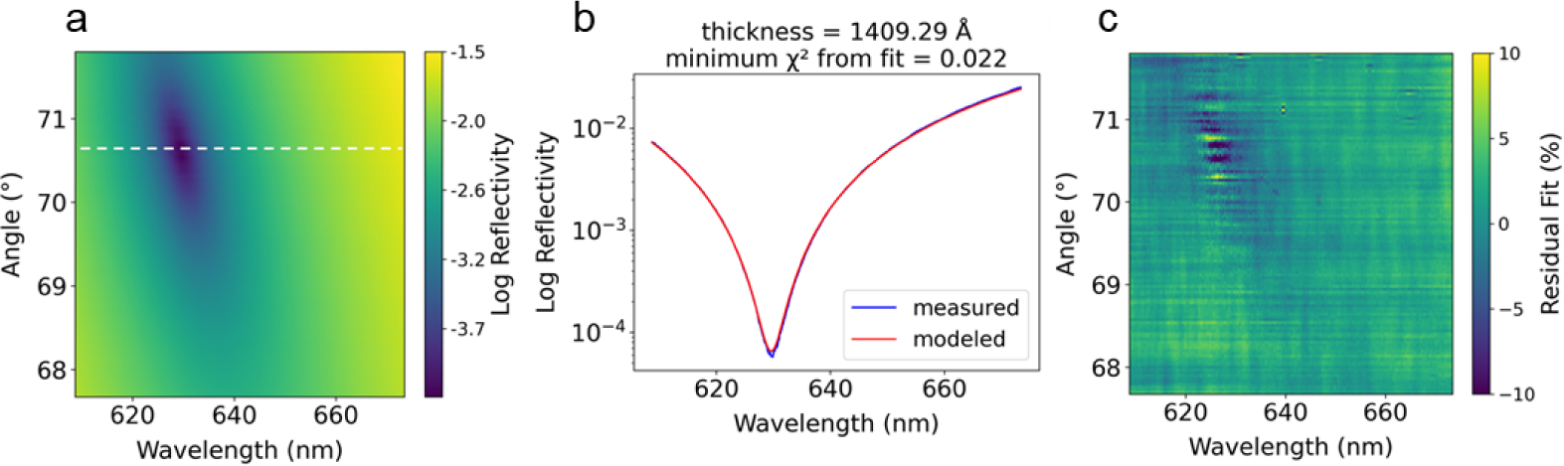
(a) Experimental LTR image with a lineout through the null reflectivity condition. (b) Reflectivity vs wavelength for measured and modeled data at the minimum fit FOM. (c) Residual fit % between the measured and modeled reflectivity data at the minimum FOM.

We investigated the substrate thickness variation using atomic force microscopy (AFM) to profile a 4 μm^2^ region of a Si/SiO_2_ substrate (Figure S1). Power spectral density analysis of the AFM micrograph identified 2-nm variations in surface height at length scales comparable to LTR illumination wavelengths, which could cause a superposition of reflectance properties on the sensor. We simulated this by applying a moving average to the theoretical reflectance data that spanned 2 nm of thickness values, and the resulting modeled reflectance was similar in magnitude to the measured values. Surface roughness at length scales much less than 600 nm, such as close-packed probe molecules, would be sampled by the light as a single thickness and would not affect the null depth or measurement integrity.

It is likely that scattered and diffracted light introduced from the optical and mechanical components in the instrument contributed to the background level of light present in the LTR images. Since it is impossible to know the extent to which each of these factors affected the depth of the reflectivity null, we chose to account for the deviation in null depth from the theoretical zero by adding the minimum reflectivity in the measured data to all points in the modeled data. This approach produced good fits without making assumptions about the magnitudes of each contributing factor.

The measured thickness was determined by identifying the parameters that minimized the fit figure of merit (FOM, eq 1).

1 $$\mathrm{FOM}=\mathrm{GWF}\times \sum \frac{{\left(R_{\mathrm{measured}}-R_{\mathrm{modeled}}\right)}^{2}}{R_{\mathrm{modeled}}}$$

The fit FOM is based on Pearson’s Chi-square statistic, a normalized Method of Least Squares that ensures that larger reflectivity values do not dominate the fit, which is important for locating minimum reflectivity. To further improve the fit, a 2D Gaussian weighting function centered on the reflectance null is applied to increase the influence of values closer to the minimum (eq 2), where *L* and *Q* represent the widths of the major and minor axes of the elliptical Gaussian, and θ determines the rotational orientation of the weighting function, *x*_0_ is *x*_min_, and *y*_0_ is *y*_min_.

2 $$\mathrm{GWF}=e^{-\left({\left(\frac{\left(x-x_{0}\right)\cos\theta -\left(y-y_{0}\right)\sin\theta }{\sqrt{2}L}\right)}^{2}+{\left(\frac{\left(x-x_{0}\right)\sin\theta -\left(y-y_{0}\right)\cos\theta }{\sqrt{2}Q}\right)}^{2}\right)}$$

This method produced good agreement between the theoretical and measured data over the full measurement range (Figure 3c). This fitting procedure leveraged the full slope of the steep null reflectivity condition and was more robust than a simple peak finding algorithm. Importantly, data points that sample the reflectance properties around the null contain valuable information that can be used to uniquely identify the thickness, even when the depth of the observed null is limited by scattered light or substrate roughness. Shifts of 1 Å, 0.1 Å, and 0.01 Å in modeled thickness increase the fit FOM by 634%, 6%, and 0.02%, respectively (Figure S2).

The performance of the LTR instrument was evaluated by measuring 35 individual substrates with SiO_2_ film thicknesses ranging from 1390 to 1465 Å by using both LTR and spectroscopic ellipsometry. This film thickness range represents the range of unfunctionalized Si/SiO_2_ substrates that we had on hand and does not cover the full 100 Å range that this LTR prototype was designed to measure. The average difference between LTR and ellipsometry SiO_2_ thickness measurements was 0.03%, and a linear regression returned a slope of 0.997 and a *y*-intercept of 3.72 (Figure 4). This demonstrates impressive agreement between two independent thickness measurement techniques, both of which have their own degree of measurement uncertainty. The uncertainty reported by the J.A. Woollam software with each ellipsometry measurement was ∼0.2 Å. The measured LTR thickness deviation of 20 repeat measurements at a single location on a single substrate was 0.01 Å. Overall, LTR was accurate to within 1% of ellipsometry measurements, with a 3-sigma precision of 0.03 Å.

**Figure 4:**
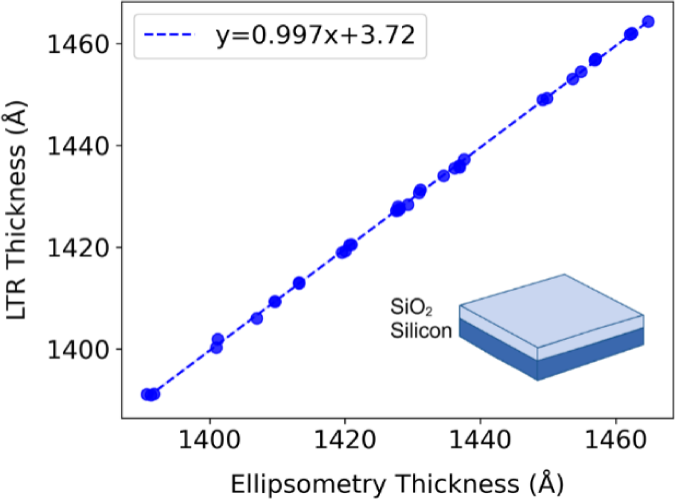
Comparison of the measurements of 35 substrates with both LTR and ellipsometry.

We examined the ability of LTR to measure the thickness of protein films formed on the entire surface of a Si/SiO_2_ substrate. LTR measurements of a 150 μm × 50 μm area were conducted on separate substrates at different steps during the silane functionalization (3-glycidyloxypropyl trimethoxysilane (GPTMS), adhesion layer, Figure 5a), probe deposition, and analyte binding. There was a 40 Å thickness increase due to the covalent attachment of the IgG molecule to the GPTMS amine-reactive adhesion layer (Figure 5b). The binding of the antibody to the IgG-coated substrate caused an additional 35 Å increase in thickness (Figure 5c). These measurements of a monolayer of protein demonstrate the movement of the null reflectivity condition in lambda–theta space as a function of optical thickness and confirm the ability to observe changes in protein films due to antibody–antigen binding. This also demonstrates the biosensing dynamic range of LTR. We used high concentrations of IgG and anti-IgG (0.5 and 3 μg/mL, respectively) for these experiments. Therefore, these protein films can be considered as a closely packed monolayer of proteins on the surface of the substrate. The ∼75 Å total thickness change due to anti-IgG and IgG proteins is less than 6% of the thickness of the underlying SiO_2_ film (∼1400 Å). Therefore, we chose to approximate the protein film layer as an extension of the SiO_2_ layer in the characteristic matrix model. This is appropriate for biosensing applications where physical thickness is less important than relative signal change due to the binding of the analyte to the probe.

**Figure 5:**
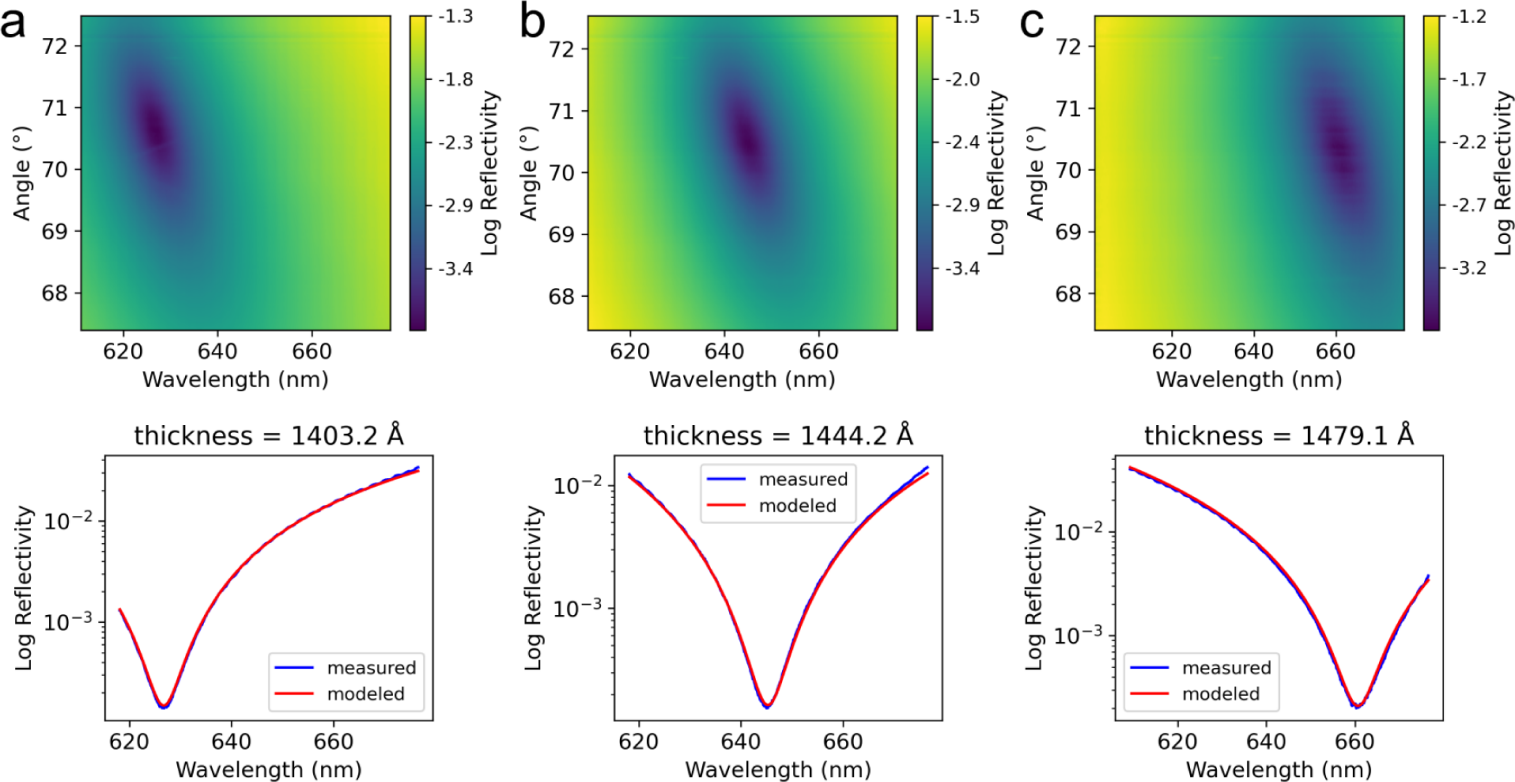
Biosensing capability of LTR with whole chip chemical and protein films. (a) LTR measurement of SiO_2_/Si substrate coated with amine-reactive silane. (b) LTR measurement of SiO_2_/Si substrate coated with amine-reactive silane and incubated in 0.5 μg/mL human IgG protein. (c) LTR measurement of SiO_2_/Si substrate coated with amine-reactive silane, incubated in 0.5 μg/mL IgG protein, and incubated in 3 μg/mL anti-IgG. In each instance, a 150 μm x 50 μm area was measured.

To examine the variation in thickness across the substrate, we measured 24 different areas of a GPTMS-treated substrate incubated in a 1% solution of bovine serum albumin (BSA), followed by a 20% solution of fetal bovine serum (FBS) in accordance with our standard blocking protocol. This substrate was not arrayed with any probes. LTR measurements were collected every 1 mm in the “*x*” dimension and every 0.5 mm in the “*y*” dimension by moving the stage holding the substrate between each measurement. We found that the thickness across a 2 × 3.5 mm area of the substrate varied over a 2 Å range with a standard deviation of 0.56 Å (Figure S3). The impact of this baseline thickness variation can be minimized by measuring neighboring control probes and using them as a correction factor in multiplexed arrays.

We next confirmed that LTR thickness measurements can be used to measure the analyte concentration. A standard curve was constructed by arraying several substrates with anti-IL-6 analyte probes and anti-fluorescein isothiocyanate (anti-FITC) control probes 300 μm apart and incubating each substrate in different serially diluted concentrations of the analyte interleukin-6 (IL-6) overnight to allow the system to reach equilibrium. IL-6 is a 26-kDa protein that modulates cell-signaling pathways in the human immune system.^19^ It is normally present in human serum at very low concentrations less than 10 pg/mL. An elevated concentration (10s to 100s of pg/mL) of IL-6 in serum is a common biomarker of inflammation and a heightened immune response.^20^ Fluorescein isothiocyanate (FITC) is a molecule that is normally not present in human samples, so we use an antibody against FITC as a control probe to account for inter- and intrasubstrate differences in the thickness of the underlying oxide layer. This is an unconjugated antibody, and no fluorescent molecule is used. After several substrates were arrayed with the anti-IL6 analyte probes and anti-FITC control probes, each substrate was fully submerged in assay solutions. The control substrate was exposed only to the complex assay diluent (salt buffer containing detergent, 1% bovine serum albumin (BSA), and 20% fetal bovine serum (FBS)) and was used to establish baseline thickness. It is the blank measurement. The analyte substrates were exposed to different concentrations of IL-6 mixed with the same assay diluent. The presence of FBS in the assay diluent makes this complex matrix comparable to a 1:5 dilution of a serum sample. Single LTR measurements were made at the center of one anti-IL-6 and an adjacent anti-FITC probe spot on each substrate.

The lower limit of detection (LLOD) is defined as the concentration of an analyte at which the sensor measures a response that is significantly different from that of the blank. The limit of the blank (LOB) was calculated from the control values (eq 3), where μ is the mean and σ is the standard deviation. The LLOD was calculated from a combination of all low-concentration thickness values and standard deviations (eq 4).^21,22^

3 $$\mathrm{LOB}=\mu_{\mathrm{blank}}+1.645\left(\sigma_{\mathrm{blank}}\right)$$

4 $$\mathrm{LLOD}=\mathrm{LOB}+1.645(\sigma_{\mathrm{low}\;\mathrm{concentration}\;\mathrm{samples}})$$

We calculated the LLOD of label-free IL-6 detection using a four-parameter logistic regression (4PL) fit to relate anti-FITC-subtracted LTR thickness measurements to IL-6 concentration (eq 5 and Figure S4), where *a* is the minimum response, *b* is the Hill slope of the curve, *c* is the point of inflection, and *d* is the maximum response. The 4PL regression fits ligand-binding response better than a 1:1 Langmuir-binding isotherm in assays that deviate from perfectly independent binding events of analytes that are monovalent and homogeneous^23^ and it generally outperforms other models in fitting the full range of response data comprising the sigmoidal shape of biological binding assays.^24^

5 $$y=d+\frac{a-d}{1+{\left(\frac{x}{c}\right)}^{b}}$$

The calculated LLOD of 539 pg/mL (0.02 nM) is at the upper limit of the clinically relevant range for IL-6 in human serum, indicating that this assay in its current form would not be useful for the measurement of IL-6 in blood samples. Commercial cytokine detection platforms rely on signal amplification using labeled molecules, and a direct comparison of label-free detection using LTR with those platforms would be inappropriate. Instead, we evaluated the theoretical and experimental limits of thickness-based detection and considered whether the LLOD of label-free detection is limited by the instrument or by assay constraints. It is possible to use sandwich antibodies and mass-based amplification techniques with LTR to improve the LLOD, but that is beyond the scope of this work.

Biological limitations include probe molecule packing density/orientation and binding pair affinity. Since the probe molecules are deposited as a liquid droplet and attach to the substrate via amine-reactive chemistry, the orientation of the probes is random, and the binding site is likely inaccessible for a portion of probes. To better understand our experimental conditions, we compared the theoretical and experimental thickness at maximum binding using the 1:1 one-site Langmuir-binding isotherm model.^17,25^ This model describes the fractional coverage of available binding sites as a function of analyte concentration and is grounded in the principles of molecular adsorption at a planar surface (eq 6).

6 $$\Gamma =\frac{C}{C+K_{D}}$$

Here, Γ is the fraction of probe sites bound by analyte, the dissociation constant (*K*_D_) is the concentration at which half of the binding sites are occupied, and *C* is the molar concentration of the analyte. The system must be at dynamic equilibrium for this model to apply, and this assay was performed overnight at 4 °C to allow it to reach equilibrium.

Experimental thickness change measurements were calculated by subtracting the anti-FITC-corrected thickness values on the control substrate (incubated only in diluent) from the anti-FITC-corrected thickness values on the analyte substrate (eq 7).

$$\mathrm{Thickness}\;\mathrm{Change}\;(Å)=\left({\mathrm{Probe}}_{\mathrm{Analyte}}-{anti\mathrm{‐FITC}}_{\mathrm{Analyte}}\right)-\left({\mathrm{Probe}}_{\mathrm{Control}}-{anti\mathrm{‐FITC}}_{\mathrm{Control}}\right)$$ 7

The 1:1 Langmuir-binding isotherm model can then be applied to assess both the experimental maximum height (*Thickness change*_*max*_) and the binding affinity (*K*_D_) of this anti-IL-6 and recombinant IL-6 binding pair (eq 8).

8 $$\mathrm{Thickness}\;\mathrm{change}=\mathrm{Thickness}\;{\mathrm{change}}_{\max}\times \frac{C}{C+K_{D}}$$

We calculated a theoretical maximum thickness change range of 5.5 ± 3 Å, which represents probe-binding site availability ranging from 40 to 100% (Supporting Information). The experimental *Thickness change*_*max*_ from the Langmuir fit was 6.4 Å (Figure 6), which corresponds to 88% availability of probe-binding sites according to the theoretical model. This is quite good, considering that these probes were randomly oriented on the surface of the substrate. This probe may not benefit from orienting techniques^26^ to increase the available binding sites, but if such an approach were used, it would not interfere with the LTR measurement or decrease null depth if applied densely enough that the length scale of the height variation is much smaller than the wavelengths of light employed by LTR.

**Figure 6:**
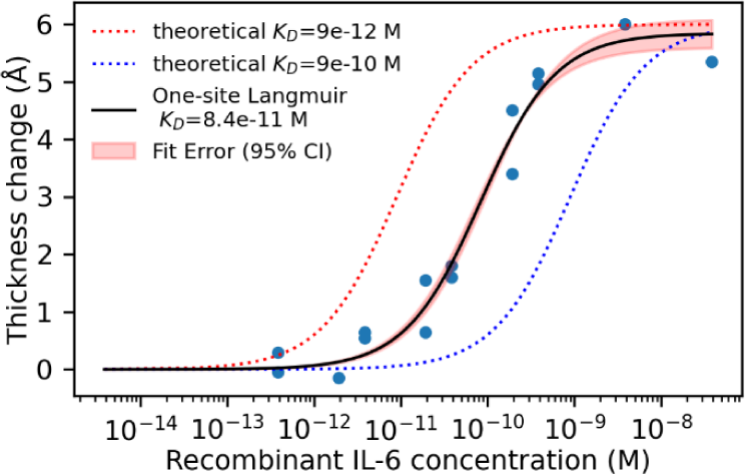
LTR measured thickness change relative to control substrate exposed only to a diluent fit with a one-site Langmuir-binding isotherm. An increase in binding affinity (a decrease in *K*_D_) shifts the isotherm to improve detection at lower concentrations.

The Langmuir fit returned an experimental *K*_D_ of 8.4E–11 M. We then assessed the impact that a 10-fold increase or decrease in *K*_D_ would have on the binding isotherm and the assay sensitivity at low analyte concentrations (Figure 6).

A lower *K*_D_ represents a stronger affinity between the analyte and probe, which means more analyte remains bound to the probe in equilibrium-binding conditions at lower concentrations, presumably increasing the measurable signal at low concentrations. We further considered the effect of the binding pair *K*_D_ on the assay sensitivity by calculating the expected LTR thickness change at several *K*_D_ values for 1 pg/mL of analyte (Table 1). The inability to detect a 0.0025 Å thickness change at 1 pg/mL for this IL-6 binding pair (*K*_D_ = 8.4E-11) is consistent with the 3σ resolution limit of the instrument (0.03 Å). If we could employ a higher affinity probe (*K*_D_ = ≤6E-12 M) in this assay, then the thickness change due to 1 pg/mL IL-6 could be measured with LTR, assuming no other sources of variation. However, differences between biological replicates would likely continue to limit the LLOD because this noninstrumental source of error has a significant contribution to the LLOD calculation. Furthermore, a more localized correction strategy may need to be employed to account for the error introduced by the intrasubstrate thickness variability, as discussed earlier. These are all assay limitations, not instrument limitations.

**Table 1:** Theoretical Thickness Change Required to Measure 1 pg/mL Analyte at Different Binding Pair (Probe and Analyte) *K*_D_ Values^a^

*K*_D_	9E-14	9E-13	4E-12	5E-12	6E-12	7E-12	8E-12	9E-12	9E-11	9E-10
Calculated thickness change (Å) at 1 pg/mL of IL-6	1.720	0.237	0.076	0.057	0.046	0.033	0.029	0.0246	0.0025	0.0002

a If the LTR thickness change resolution of 0.03 Å was the only factor governing the LLOD, then the assay could detect the thickness change due to 1 pg/mL of IL-6 binding to probe when the binding pair *K*_D_ is ≤6E–12 M.

The motivation for designing and constructing this LTR instrument was a desire to overcome the errors of AIR measurements when the initial probe thickness is not optimized to the antireflective baseline condition. To demonstrate the improvement that LTR offers over AIR, we compared measurements of an array of probes rejected from a previous AIR study because it was fabricated on a “too thin” substrate. This array consists of 21 proteins produced by (6 replicate spots of each) and 71 replicate anti-FITC correction probes around the border (Figure S5). The proteins are found on the surface of the bacteria or are secreted into the environment and are known to have immunomodulatory effects,^27^ including the production of anti- antibodies with lock-and-key specificity. A version of this multiplex array (built on substrates with optimal baseline thickness for AIR) was used in previous work to quantify the human antibody response to bacteria in the contexts of infection or related disease.^28,29^ In brief, the array consisted of proteins representing 6 broadly defined functional classes:

Iron acquisition proteins (iron-regulated surface determinant proteins IsdA, IsdB, and IsdH).
Cell division and cell wall proteins (autolysin domains glucosaminidase [Gmd] and amidase [Amd], immunodominant staphylococcal antigen A [IsaA])
Biofilm formation proteins (clumping factor A [ClfA] and bone sialoprotein-binding proteins [BsBp])
Immune evasion proteins (chemotaxis inhibitory protein of [CHIPS]; staphylococcal complement inhibitory protein [SCIN])
Superantigens (staphylococcal enterotoxin A [SEA], B [SEB], and C [SEC]; staphylococcal enterotoxin-like G [SE*l*G], I [SE*l*I], Q [SE*l*Q], X [SE*l*X], and toxic shock syndrome toxin-1 [TSST-1])
Cytotoxins (leukocidin F [LukF] and S [LukS], and alpha-hemolysin [Hla]).

As seen in the AIR image of the substrate incubated with only diluent (Control), many probes were not at the optimal baseline thickness for AIR. A handful of probes decreased in AIR reflectivity upon incubation with 1:250 diluted serum from an individual with a culture-confirmed *S. aureus* infection (analyte), which indicates that they are too thin for accurate thickness measurements using AIR. (Figure 7a). This is problematic because the calibration curve of AIR versus thickness is a parabola and cannot return a unique thickness value (Figure 7b). Since AIR relies on the optimization of baseline probe thickness to the antireflective condition, the technique defaults to the positive root for reflectivity-to-thickness conversions. Therefore, too-thin probes to the left of the minimum will decrease in reflectivity as analyte binds, and AIR will return a negative thickness change. In the scenario shown by the X, a decrease in reflectance could correspond to either a 7 or 17 Å build. No change in reflectance could correspond to either a 0 or 25 Å build. We were able to use LTR to measure the thickness of each probe on the control substrate (Figure 7c). This is the first time that we have been able to accurately measure the baseline probe thickness on these “too-thin” arrays. We measured the thickness change after analyte bound to the “thin” probes with both AIR and LTR. LTR measurements for each probe type are shown in Figure S6. A comparison of the AIR and LTR thickness change measurements for the thin probes is shown in Figure 7d. These are measurements of a single probe on each substrate and do not have replicate variability to report. As expected, AIR returned negative or no changes in thickness for these probes that started at thicknesses to the left of the reflectivity minimum. LTR was able to rescue the functionality of these probes and produce robust measurements of thickness change due to bound antibody. The highest increase in thickness due to antibody binding was observed for the SEA, SEC, Se*l*I, and SE*l*Q antigens, which were the thinnest probes on the control array. The ClfA and BsBp response is compelling because these probes were increasing in thickness but not changing in reflectivity using AIR. They were “moving across the parabola” from the left to the right.

**Figure 7:**
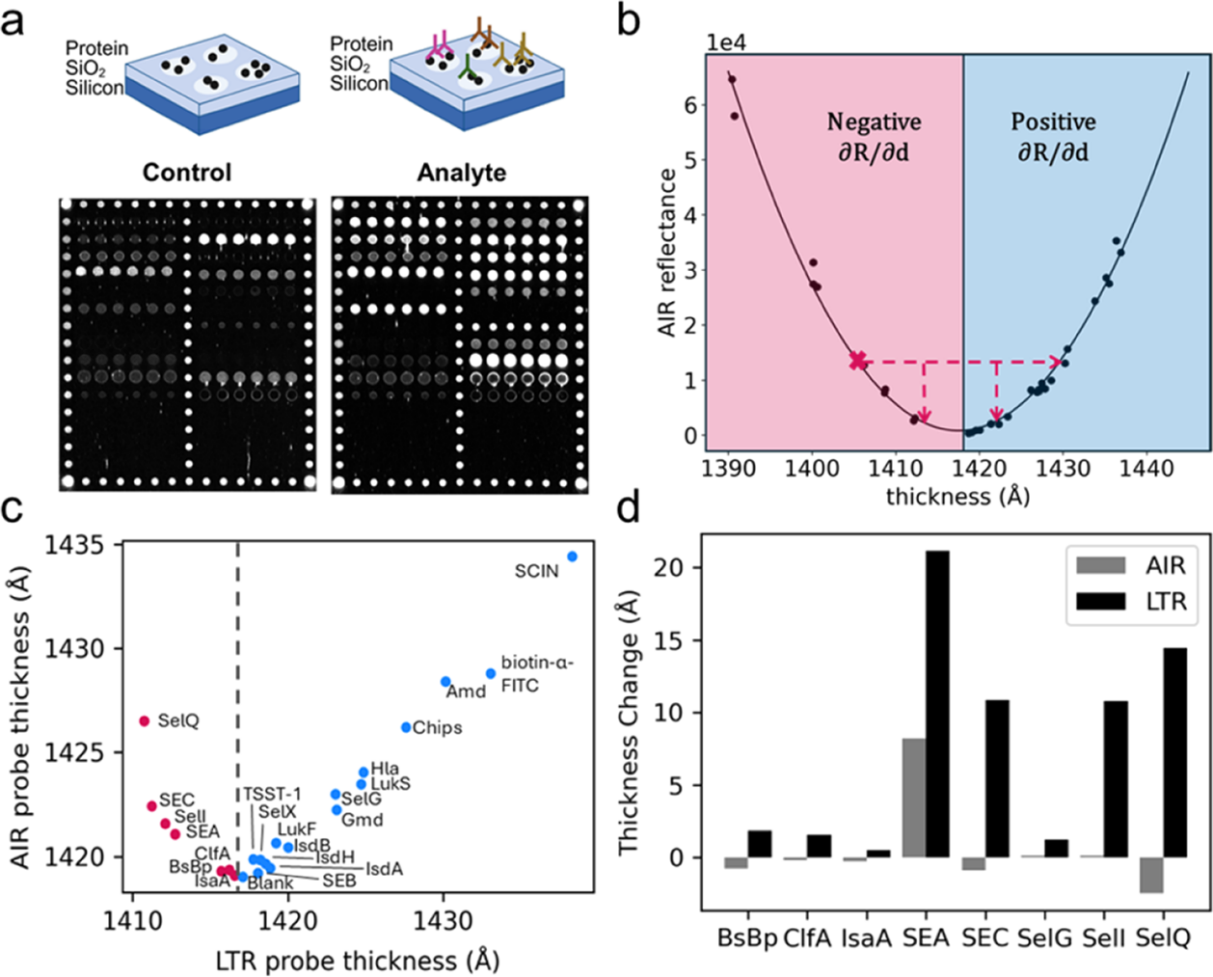
LTR measured the thickness of each probe on a multiplex antibody detection array and rescued probes that were too thin for accurate AIR binding measurements. (a) AIR images of control and analyte substrates. The control substrate was incubated in the assay diluent. The analyte substrate was incubated in serum from an individual with a culture-confirmed infection. (b) AIR determines thickness measuring reflectivity at a single wavelength and AOI. The AIR reflectivity vs thickness curve is parabolic and does not provide unique thickness measurements. When the starting thicknesses are below the minimum reflectivity, thickness build can result in either a drop in reflectivity or possibly no change in reflectivity at all. The positive root is assumed by default and returns negative thickness changes for probes that are too thin. (c) LTR provides accurate thickness measurements for all probes on the control chip. (d) AIR returned negative thickness change, no change, or weak positive changes for probes that were too thin. LTR rescued these probes and provided accurate thickness change measurements, resulting from antibodies bound to the proteins on the array.

## Conclusions

The key technical advancement of LTR is the simultaneous illumination of a thin-film substrate over a range of wavelengths and AOIs with the ability to resolve the angular and spectral content of the reflected light along two orthogonal dimensions concurrently. This enables LTR to identify the null reflectivity condition over a range of film thicknesses without scanning through the angle or wavelength.

LTR fundamentally measures optical thickness. For an absolute physical thickness measurement, an external measurement of the refractive index would be required. This is not necessary when using LTR as a biosensor since the metric of interest is thickness change due to analyte binding to probe molecules, and a relative thickness measurement is appropriate. Furthermore, the protein refractive index varies according to the illumination wavelength, as well as amino acid content, concentration, temperature, and extent of hydration,^30^ which would be difficult to measure and model for each analyte and probe on the array.

The thickness resolution of the LTR instrument itself may be enhanced by deepening the null reflectivity condition. We assessed several sources of noise in the system, including scattered light, temperature effects, and other factors affecting finite resolution limits. The variation in the repeated measurements appears to be a function of the temperature changes and lamp output fluctuations over the course of the measurement window. Localized heating at the illumination region, as well as increased ambient temperature in the instrument box from heat dissipated by the sensor electronics, could cause changes in the optical thickness of the film over time. Physical layer thickness and material index of refraction are both temperature dependent, which can lead to drifts in the measured optical thickness.

While the ability of LTR to measure protein binding on planar arrays is encouraging, we have considered avenues for further improvement. The sensitivity at low concentrations could be enhanced by selecting probes with higher affinity (lower *K*_D_) for the analyte of interest. Another approach would be to include a sandwich antibody or other mass-based amplification strategies to increase the thickness at low analyte concentrations. The measurement sensitivity also depends on the variation between biological replicates because the standard deviations of the blank and low-concentration samples are fundamental to the LLOD calculation. There are differences in base oxide thickness both between control and analyte chips, as well as across the surface of a single chip. Hyperlocalized corrections for baseline thickness variations could decrease the variability between replicates. This would either mean using an adjacent anti-FITC probe or sampling the background area immediately next to each analyte probe for inter- and intrachip corrections rather than using only a single anti-FITC measurement or averaging values across a substrate.

It is important to consider that ultralow LLODs are not the only metric for evaluating biosensor performance. Other considerations include ease of use, accuracy, multiplexing capability, and dynamic range, all of which are strengths of LTR. The Si/SiO_2_ substrate is large enough to fit hundreds of probes, and the 100 Å measurement range eliminates the need for multiple sample dilutions. While LTR requires scanning of the substrate to image an entire array (unlike AIR, which acquires a single image of the entire substrate), programmable translational stages could advance this technique beyond the prototype stage and toward higher throughput use. We also considered another high-throughput design that can measure several probes on several substrates simultaneously. However, the work presented here is meant to be a proof of concept for the LTR approach to biosensing. To this end, this prototype LTR instrument measured SiO_2_ film thickness with accuracy within 1% of ellipsometry and demonstrated impressive 0.01 Å precision. It measured thickness changes due to protein-binding events over a 100 Å range, representing concentrations of <1 ng/mL for the small IL-6 protein to 3 μg/mL for larger IgG and anti-IgG proteins, and anti- antibodies in human serum. Other studies have shown that specific antibodies against proteins are present at concentrations greater than 1 μg/mL in human serum.^31^ This large sensing range allows multiple protein types to be measured simultaneously from a single sample dilution. Furthermore, the sensing range of LTR is controlled by the range of wavelengths and angles present in the illumination, which can be expanded if needed.

We developed LTR to address the challenging workflow of current reflectometric methods, particularly the stringent requirements for baseline probe thickness to achieve the antireflective condition when using AIR. LTR was able to recover antibody-binding information from a antigen array that was not optimized for AIR. The small sample region and sub-Ångström precision of LTR combined with a straightforward data-reduction technique provide a simpler tool for biosensing than ellipsometry.^32–34^

The prototype device used in this report returns a single thickness measurement and does not require precise tuning of the baseline probe thickness, which enables highly accurate measurements with relaxed array manufacturing requirements. This will be particularly helpful in our ongoing work studying the antibody response to infection. Other potential applications include the study of autoimmune,^35^ neuroinflammatory,^36^ and infectious diseases.^37^ Biomarker panels are typically more effective at diagnosing diseases than single-plex assays,^38^ and they provide more information from a single sample in scientific studies. LTR could be used to measure concise panels consisting of biomarker antigens, cytokines, and antibodies. This type of “mixed assay” is possible because LTR is a label-free measurement, and the sensing range spans several logs of concentration while requiring only a single sample dilution. The ability to make accurate measurements of multiplexed biological arrays while loosening the probe deposition requirements enables us to use this technique to study a wider set of biomarkers.

## Materials and Methods

### LTR Instrument Calibration

The LTR optics were arranged to sort and direct photons to specific locations on the detector plane based on their wavelength and angle of incidence at the biosensor surface. Wavelength calibration was established using a Neon Spectral Calibration Lamp that produces 15 detectable emission lines spanning from 607 to 671 nm. Each of these lines provided a unique wavelength position and spectral resolution measurement at a different location across the image. The spectral resolution varies over the field of view and ranges from 0.6 to 1.0 nm full width at half maximum. The optical design optimized spectral resolution by balancing the angular width of the slit aperture with the spread of angles introduced by diffraction as light passed through the slit. Wavelength values at pixel positions between calibration points were inferred using cubic spline interpolation.

A value for the angular span across a single pixel (Δθ/Δpixel) was established by locating the edges of the illumination field and relating the cutoff positions on the image to the angular extent of the system aperture stop. A pinhole placed in the center of the optical axis generates a setup image that identifies the 70° reflectance AOI based on the geometry of the nominal optomechanical design. Together, this information was used to generate a linear relationship between the AOI and pixel position. In practice, this angular calibration technique was used as a starting point for analysis and was later refined empirically by varying the values (±5%) to improve the goodness-of-fit to the LTR data. This is possible because locating the null position in wavelength space uniquely identifies the substrate thickness and the corresponding null AOI. The absolute accuracy of the wavelength calibration is comparably well-known, allowing the angle calibration to be adjusted during analyses.

Absolute reflectance measurements for each pixel in the LTR image were made by first recording a flat-field calibration that baselines the relative signal levels using a substrate with a known reflectance. A Si/SiO_2_ substrate coated with 300 nm of aluminum and 3 nm of SiO_2_ by E-beam physical vapor deposition served as a reflectance standard with nominal reflectivity, *R*_FF_(λ,θ), calculated from first principles. The signal present in a given pixel, ADU_*i*_(*x*,*y*) was determined by eq 9, where *P* is the lamp emission given in photons/second, *R* is the reflectivity of the substrate, *T* is the system transmission, including diffraction and optical coatings, QE is the quantum efficiency of the detector (CCD*e*–/photon), *G* is the camera gain given in (ADU/CCD*e*−), and *t* is the image exposure duration.

9 $${\mathrm{ADU}}_{\mathrm{FF}}\left[x,y\right]=P\left(\lambda ,\theta \right)\times R_{\mathrm{FF}}\left(\lambda ,\theta \right)\times T\left(\lambda ,\theta \right)\times \mathrm{QE}\left(\lambda \right)\times G\times t_{\mathrm{FF}}\times \Delta \lambda \times \Delta \theta$$

A similar expression determined the signals observed when a functionalized Si/SiO_2_ chip was used. The absolute reflectance of the LTR data was calculated by using a ratio of these two images. Assuming the instrument sensitivity parameters and lamp output power remain constant over time, many parameters cancel, and the reflectance is given by eq 10:

10 $$R\left(\lambda ,\theta \right)=\frac{{\mathrm{ADU}}_{\mathrm{LTR}}\left[x,y\right]\times t_{\mathrm{FF}}\times R_{\mathrm{FF}}\left(\lambda ,\theta \right)}{{\mathrm{ADU}}_{\mathrm{FF}}\left[x,y\right]\times t_{\mathrm{LTR}}}$$

An external shutter controls the lamp exposure duration and is adjusted such that the sensor well capacity is nearly full in the brightest regions of the image. Typical exposure durations are 200 ms for flat-field calibrations and 10 s for LTR images.

### Substrate Preparation

Polished 300-mm silicon wafers with 140 to 150 nm of thermally grown silicon dioxide were fabricated by SUNY Polytechnic Institute, diced into 5 × 5.7 mm substrates, and binned by oxide thickness according to measurements made by a J.A. Woolam M2000 ellipsometer. Substrates were taken from a range of binned thicknesses, cleaned in piranha solution (3 parts sulfuric acid to 1 part 30% hydrogen peroxide) for 30 min, washed 3 times in Nanopure water, and dried with a stream of N_2_ gas prior to LTR measurements. The substrates used for protein measurements were selected to have similar oxide thickness and washed to remove dicing debris (7:3 solution of ethanol and 10 M NaOH) for 30 min, then etched in hydrofluoric acid to reach ∼1400 Å, rinsed with water, and dried with N_2_ gas before being chemically functionalized with ∼5 Å of amine-reactive (3-glycidyloxypropyl) trimethoxysilane (GPTMS (Sigma-Aldrich 440167)) in a plasma process chemical vapor deposition system (YES 1224P) at the University of Rochester Integrated Nanosystems Center.

### Protein Films across Entire Substrate Surface

Surface protein films were grown by incubating entire substrates overnight at room temperature in a 0.5 μg/mL solution of human IgG whole molecule protein (Rockland 009-0102) and phosphate-buffered saline (PBS: 137 mM NaCl, 2.7 mM KCl, 10 mM Na_2_HPO_4_, 1.76 mM KH_2_PO_4_·H_2_O, pH 7.4) at 300 rpm on an orbital shaker. Substrates were next placed in assay wash buffer (AWB: mPBS with 0.005% Tween-20, pH 7.2) for 5 min before incubating in 3 μg/mL of anti-IgG protein (Rockland 609-701-123) solution in PBS for 1 h at room temperature. Substrates were removed from each step of this process for LTR measurements. All substrates were washed with Nanopure water and dried with a stream of N_2_ gas before being measured.

### AFM Measurements

A NT-MDT AFM microscope was used at the University of Rochester Integrated Nanosystems Center in semicontact tapping mode at a 10 μm/second scan rate over a 5 μm^2^ area. Image postprocessing and power spectral density analysis were accomplished using open-source Gwyddion software.^39^

### Arraying Protein Probes onto Substrates

Substrates with arrayed probes first underwent the above-described base wash, HF etch, and GPTMS functionalization process before having protein solutions deposited by an SX sciFLEXARRAYER (Scienion A.G., Berlin, Germany) using a PDC70 capillary with type 4 coating. The protein–probe mixtures comprising the microarray were pipetted into individual wells of a 384-well plate. Substrates were mounted onto adhesive strips and placed inside the SX arrayer, which was set to 85% ± 4% relative humidity. Droplets were between 300 and 350 pL in volume, as measured by the instrument. After the completion of the arraying process, the chips remained in the humidified chamber overnight to ensure the covalent attachment of the protein probes to the GPTMS-functionalized substrates.

### IL-6 Standard Curve Substrates

Anti-IL-6 (BioLegend 501125) was diluted in PBS to 400 μg/mL. Anti-FITC (Rockland 600-101-096) was dialyzed to remove sodium azide by using 20-kDa molecular weight cutoff Slide-A-Lyzer dialysis cups floating in a beaker of PBS stirred slowly with a magnetic stir bar for 1.5 h at RT before dilution in PBS to a concentration of 500 μg/mL for arraying.

###  Antigen Substrates

Recombinant antigens were selected and produced with a biotin tag as described previously.^40^ The antigen formulations are detailed in Figure S7. All probe solutions contained 2% trehalose as an additive to improve spot morphology. These arrays were originally manufactured for use with AIR, where biotin and avidin were applied in a (failed) attempt to boost baseline probe thickness. Biotin and avidin are neither necessary nor harmful for LTR measurements.

### Blocking the Background of Arrayed Substrates

All incubations were performed with stabilized microarrayed chips mounted on adhesive combs inserted into individual wells of a 96-well plate on an orbital shaker at 300 rpm. The probe-arrayed chips were removed from the humidified chamber and immediately placed into wells of a 96-well plate, each containing 300 μL of 50 mM NaOAc, pH 5.0, for 5 min to prevent the smearing of any unadsorbed probes onto nearby areas of the amine-reactive chip surface. The chips were then incubated in 1% bovine serum albumin (BSA: Rockland BSA-50) in NaOAc, pH 5.0, for 30 min to block the background from nonspecific adsorption and then transferred to wells containing a second blocking solution of 20% fetal bovine serum (FBS: Gibco A5670701) in assay wash buffer (AWB: mPBS with 0.005% Tween-20, pH 7.2) for 30 min. All incubation steps were performed at RT with orbital shaking (300 rpm).

### Analyte Incubations

The assay diluent in all cases was Enhanced Assay Buffer (EAB: 20 mM Tris Base, 250 mM NaCl, 250 mM KCl, 3% w/v propylene glycol, 0.125% Triton X-100, and 1% w/v BSA) with 20% FBS. Each substrate for the IL-6 standard curve was incubated in serially diluted (10000, 5000, 1000, 500, 100, 50, and 10 pg/mL) recombinant human IL-6 (BioLegend 570804) overnight at 4 °C shaking at 420 rpm. Substrates were then washed in AWB and Nanopure water and dried with a stream of N_2_ gas. Substrates for antibody detection were incubated in a 1:250 dilution of human serum from an individual with a culture-confirmed infection overnight at 4 °C shaking at 420 rpm, washed in AWB for 5–10 min, then exposed to a 1-h RT incubation with an Fcγ-specific anti-IgG (AffiniPure 109-005-008) to amplify the response. This anti-IgG amplification step was performed to maintain consistency with previous AIR assays conducted using similar arrays. Chips underwent a final incubation in AWB for 10 min before being rinsed in Nanopure water and dried under a stream of nitrogen gas. Control chips underwent the same process but were only exposed to the assay diluent during the primary incubation step.

### AIR Image Analysis

AIR images were analyzed in ImageJ software. Regions of interest were drawn around individual probe spots to measure the median pixel intensity. These intensity values were converted to thickness using the equation of the parabolic fit of the AIR vs ellipsometry calibration. The piranha-washed Si/SiO_2_ substrates with a range of oxide thicknesses were also measured with AIR to construct the AIR versus ellipsometry calibration curve. The AIR intensity value depends on the CCD exposure time, so the exposure time of the image was matched with the calibration curve exposure time. Care was taken to avoid measuring probe spots that were overexposed, so multiple AIR images were collected over a range of exposure times. Measurements were taken from images where the median intensity was 30 000 to 40 000 counts.

## Supplementary Material



## Notes

AFM micrograph of a Si/SiO_2_ substrate with power spectral density analysis; Fit FOM vs modeled thickness; LTR measurements across a protein-blocked Si/SiO_2_ substrate; 4-parameter logistic fit of IL-6 LTR measurements; probe locations on array; LTR antibody detection measurements for each probe on*S. aureus* array; antigen formulations; Protein binding model for LTR (PDF)

## Abbreviations

LTR: lambda theta reflectometry
OI-RD: oblique-incidence reflectivity difference
RIfS: reflectometric interference spectroscopy
BLI: biolayer interferometry
SRIB/IRIS: spectral reflectance imaging biosensor/interferometric reflectance imaging sensor
AIR: arrayed imaging reflectometry
AOI: angle of incidence
AFM: atomic force microscopy
FOM: figure of merit
GPTMS: glycidoxypropyl trimethoxysilane
FITC: fluorescein isothiocyanate
IL-6: interleukin-6
LLOD: lower limit of detection
LOB: limit of the blank
4PL: four-parameter logistic
*K*_D_: dissociation constant
IsdA,B,H: iron-regulated surface determinant protein A,B,H
Gmd: glucosaminidase
Amd: aminidase
IsaA: immunodominant staphylococcal antigen A
ClfA: clumping factor A
BsBp: bone sialoprotein-binding protein
CHIPS: chemotaxis inhibitory protein of
SCIN: staphylococcal complement inhibitory protein
SEA,B,C: staphylococcal enterotoxin A,B,C
Se*l*G,I,Q,X: staphylococcal enterotoxin-like G,I,Q,X
TSST-1: toxic shock syndrome toxin-1
LukF: leukocidin F
LukS: leukocidin S
Hla: alpha-hemolysin
PBS: phosphate-buffered saline
AWB: assay wash buffer
BSA: bovine serum albumin
EAB: enhanced assay buffer
