# Supplementary material for: Lambda Theta Reflectometry: A New Technique for Measuring Optical Film Thickness in Planar Protein Arrays
Source: ACS Sens. 2025 Jul 2;10(7):5097–107. doi: 10.1021/acssensors.5c01108 (PMC12305664; doi:10.1021/acssensors.5c01108)
Supplement: Supplementary file 1 [file se5c01108_si_001.pdf]

Supporting Information

Lambda Theta Reflectometry: a new technique to measure optical film thickness applied to planar protein arrays

Alanna M. Klose,[a,b] Joseph D. Katz,[c] Robert Boni,[c] David Nelson[c], Brian Hassard[e], Benjamin L. Miller[a,b,d,e*]

[a]*Department of Dermatology, University of Rochester, Rochester, New York 14627, USA,* [b]*Materials Science Program, University of Rochester, Rochester, New York 14627, USA,* [c]*Laboratory of Laser Energetics, University of Rochester, Rochester, New York 14627, USA,* [d]*Department of Biomedical Engineering, University of Rochester, Rochester, New York 14627, USA,* [e]*Institute of Optics, University of Rochester, Rochester, New York 14627, USA*
*Author to whom correspondence should be addressed: benjamin_miller@urmc.rochester.edu

Table of Contents

Figure S1: AFM analysis of a Si/SiO_2 substrate……………………………………………...S2

Figure S2: Fit figure of merit (FOM) vs. modeled thickness………………………………....S3

Figure S3: LTR measurements across a protein-blocked Si/SiO_2 substrate………………….…S3

Figure S4: 4-parameter logistic fit of FITC-corrected LTR thickness measurements of IL-6…..S4

Figure S5: Grid map for *S. aureus* proteins on the array……………………………………...S4

Figure S6: LTR antibody detection measurements for each probe on *S. aureus* array………….S5

Figure S7: Formulations for *S. aureus* antigens printed on the array……………………………S6

Protein binding model for LTR………………………………………………………………….S7

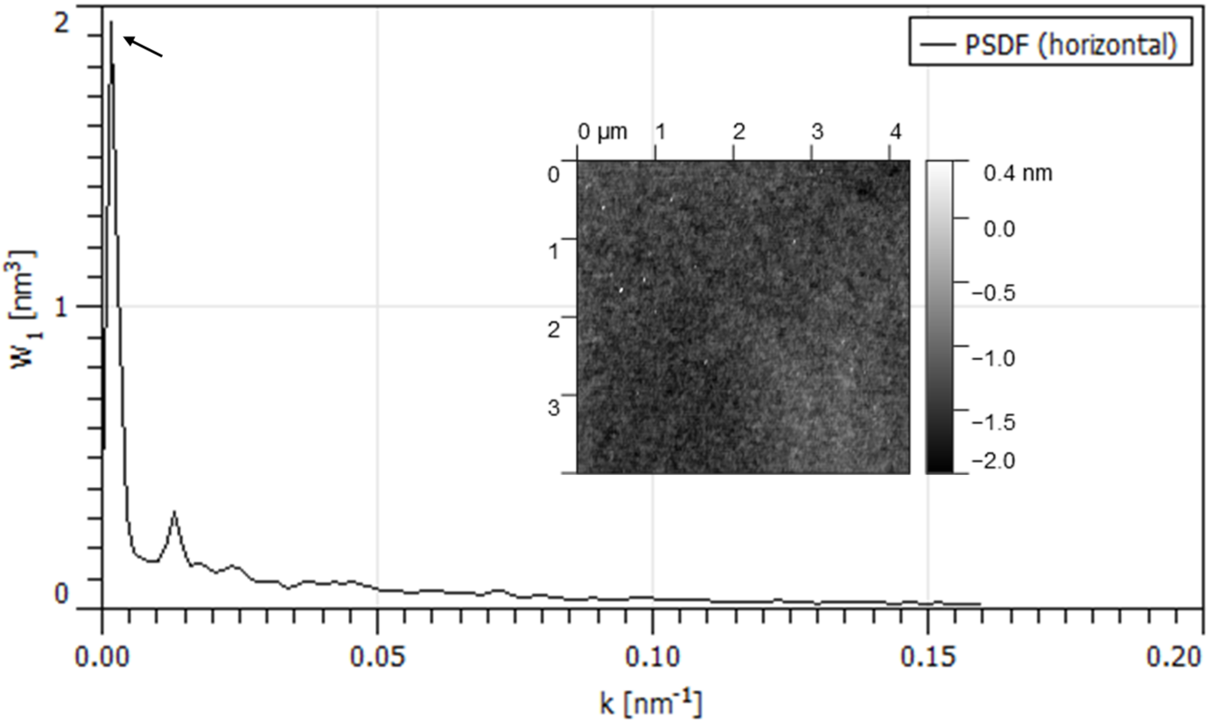

Figure S1. AFM micrograph of an ~4 µm^2 area (10 µm/second scan rate) of a Si/SiO_2 substrate. The horizontal power spectral density function shows a high frequency of height variation on a length scale of 0.0015 nm-1, or 684 nm (indicated by arrow). This frequency is larger than the wavelengths of light used by LTR and these height differences could result in the superposition of null reflectivity values.

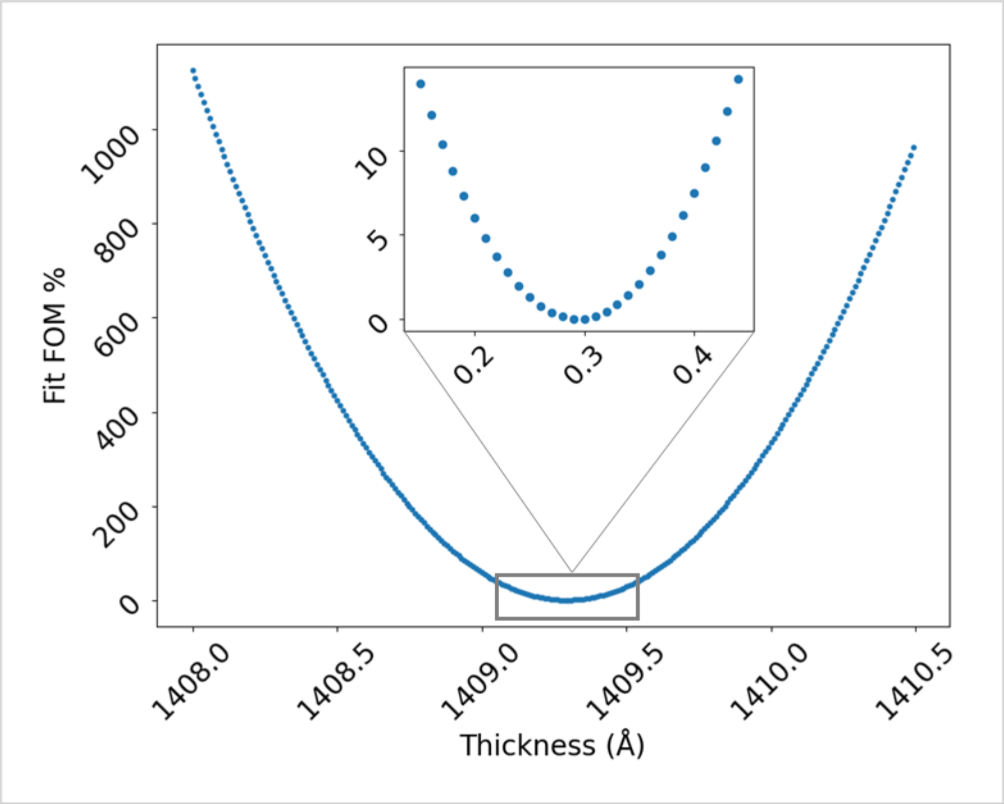

Figure S2. Fit FOM vs modeled thickness. Shifts of 1 Å, 0.1 Å, and 0.01 Å in modeled thickness increase the fit FOM by 634%, 6%, and 0.02% respectively.

y-position (mm) \ x-position (mm)	1	2	3
1	1415.31	1414.255	1413.68
1.5	1415.075	1414.29	1413.92
2	1415.015	1414.43	1414.275
2.5	1415.16	1414.825	1414.675
3	1415.31	1415.125	1414.885
3.5	1415.49	1415.32	1415.1
4	1415.57	1415.505	1415.185
4.5	1415.69	1415.65	1415.33

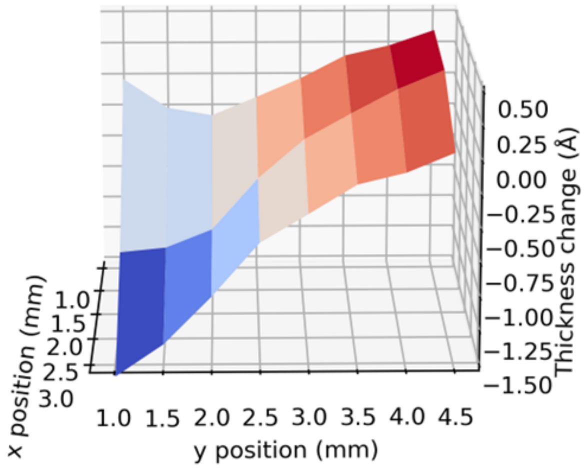

Figure S3. LTR measurements across a 2 x 4.5 mm area of a GPTMS-treated and protein-blocked substrate without any probes arrayed. The thickness ranges over 2 Å within this region and the standard deviation is 0.56 Å.

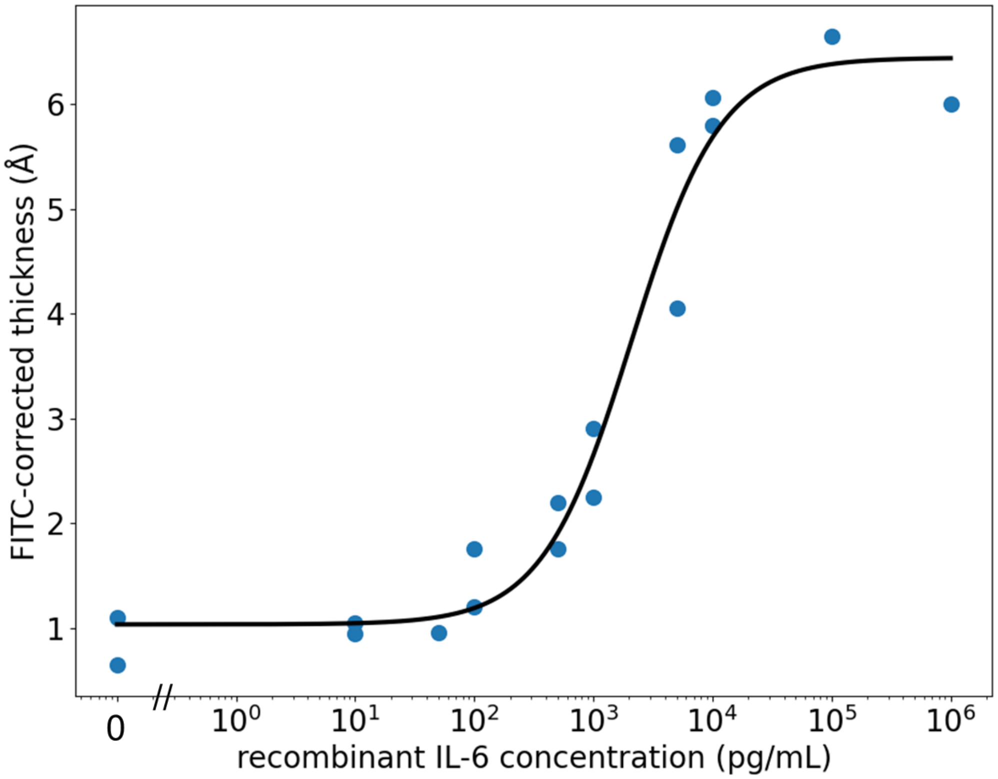

Figure S4. 4-parameter logistic fit of FITC-corrected LTR thickness measurements of IL-6. This fit was used to return the LLOD of 539 pg/mL for this assay. Measurements are plotted as individual points to show the spread of the data rather than reporting average and standard deviation of biological replicates at each concentration.

hIgG	α-FITC	α-FITC	α-FITC	α-FITC	α-FITC	α-FITC	α-FITC	α-FITC	α-FITC	α-FITC	α-FITC	α-FITC	α-FITC	hIgG
α-FITC	IsdB	IsdB	IsdB	IsdB	IsdB	IsdB	α-FITC	IsdH	IsdH	IsdH	IsdH	IsdH	IsdH	α-FITC
α-FITC	Gmd	Gmd	Gmd	Gmd	Gmd	Gmd	α-FITC	SCIN	SCIN	SCIN	SCIN	SCIN	SCIN	α-FITC
α-FITC	Hla	Hla	Hla	Hla	Hla	Hla	α-FITC	IsdA	IsdA	IsdA	IsdA	IsdA	IsdA	α-FITC
α-FITC	Amd	Amd	Amd	Amd	Amd	Amd	α-FITC	CHIPS	CHIPS	CHIPS	CHIPS	CHIPS	CHIPS	α-FITC
α-FITC	Blank	Blank	Blank	Blank	Blank	Blank	α-FITC	LukF	LukF	LukF	LukF	LukF	LukF	α-FITC
α-FITC	LukS	LukS	LukS	LukS	LukS	LukS	α-FITC	BsBp	BsBp	BsBp	BsBp	BsBp	BsBp	α-FITC
α-FITC	IsaA	IsaA	IsaA	IsaA	IsaA	IsaA	α-FITC	SEA	SEA	SEA	SEA	SEA	SEA	α-FITC
α-FITC	ClfA	ClfA	ClfA	ClfA	ClfA	ClfA	α-FITC	SEB	SEB	SEB	SEB	SEB	SEB	α-FITC
α-FITC	SEC	SEC	SEC	SEC	SEC	SEC	α-FITC	TSST1	TSST1	TSST1	TSST1	TSST1	TSST1	α-FITC
α-FITC	SelG	SelG	SelG	SelG	SelG	SelG	α-FITC	SelQ	SelQ	SelQ	SelQ	SelQ	SelQ	α-FITC
α-FITC	SelI	SelI	SelI	SelI	SelI	SelI	α-FITC	SelX	SelX	SelX	SelX	SelX	SelX	α-FITC
α-FITC	Blank	Blank	Blank	Blank	Blank	Blank	α-FITC	Blank	Blank	Blank	Blank	Blank	Blank	α-FITC
α-FITC	Blank	Blank	Blank	Blank	Blank	Blank	α-FITC	Blank	Blank	Blank	Blank	Blank	Blank	α-FITC
α-FITC	Blank	Blank	Blank	Blank	Blank	Blank	α-FITC	Blank	Blank	Blank	Blank	Blank	Blank	α-FITC
α-FITC	Blank	Blank	Blank	Blank	Blank	Blank	α-FITC	Blank	Blank	Blank	Blank	Blank	Blank	α-FITC
α-FITC	α-FITC	α-FITC	α-FITC	α-FITC	α-FITC	α-FITC	α-FITC	α-FITC	α-FITC	α-FITC	α-FITC	α-FITC	α-FITC	α-FITC

Figure S5. Grid defining the *S. aureus* proteins comprising each spot on the *S. aureus* array.

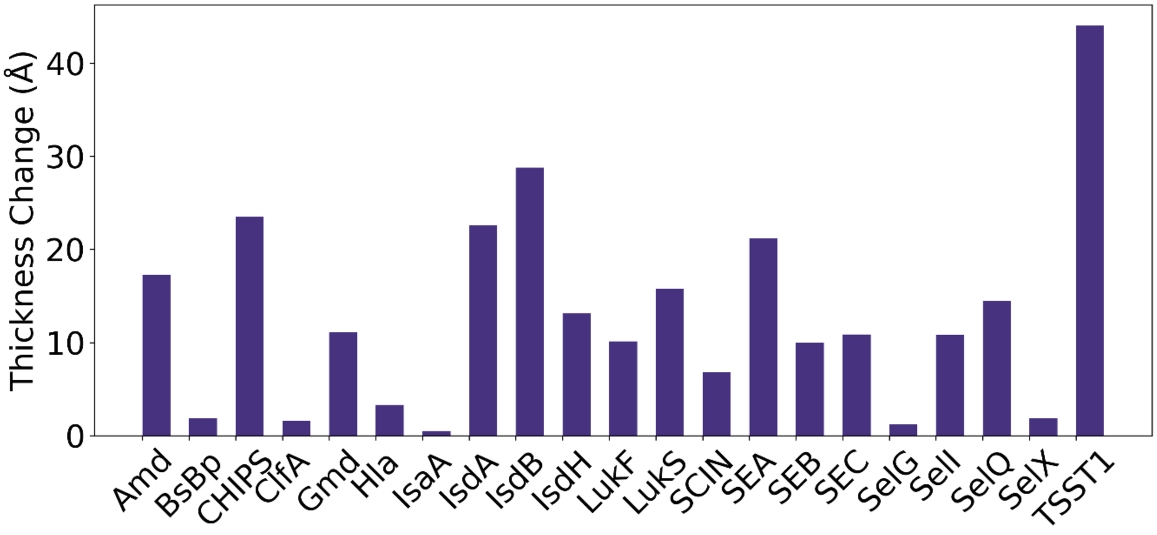

Figure S6. LTR thickness change measurements for each probe type on the *S. aureus* antibody detection array shown in figure 7. These are single measurements from the center of each probe type to demonstrate proof of concept and do not have replicate variability to report.

	# drops	Probe	Undialyzed Concentration (μg/mL)	1:4 dilution	1:8 dilution	printed conc. (μg/mL)	volume of probe (μg)	volume of 9 μM avidin (μL)	volume of 7.4 pH PBS (μL)	volume of 25% trehalose (μL)
1	1	anti-FITC biotin	1077			200	0.9	0.5	3.2	0.4
2	1	human IgG	5000			800	0.8	0	3.8	0.4
3	2	ClfA	300			120	4.1	0.5	0.0	0.4
4	2	SEA biotin	951			400	2.1	0.5	2.0	0.4
5	2	SEB biotin	954			400	2.1	0.5	2.0	0.4
6	2	SEC biotin	951			400	2.1	0.5	2.0	0.4
7	2	TSST-1 biotin	1306			800	3.1	0.5	1.0	0.4
8	2	SelG biotin	1218			900	3.7	0.5	0.4	0.4
9	2	SelQ biotin	500			410	4.1	0.5	0.0	0.4
10	2	Sel I biotin	487			200	2.1	0.5	2.0	0.4
11	1	Sel X biotin	1814			500	1.4	0.5	2.7	0.4
12	1	IsdB	4700		587.5	200	1.7	0.5	2.4	0.4
13	1	IsdH	1300			200	0.8	0.5	3.3	0.4
14	1	Gmd	1000			200	1.0	0.5	3.1	0.4
15	1	SCIN	unknown				1	1	7.2	0.8
16	1	Hla	3800	950		400	2.1	0.5	2.0	0.4
17	1	IsdA	3100	775		400	2.6	0.5	1.5	0.4
18	1	Amd	9000		1125	200	0.9	0.5	3.2	0.4
19	1	Chips	2300	575		400	3.5	0.5	0.6	0.4
20	1	LukS	3500	875		200	1.1	0.5	3.0	0.4
21	1	BsBp/SdrE	600			400	3.3	0.5	0.8	0.4
22	1	IsaA	480			390	4.1	0.5	0.0	0.4
23	1	LukF	1624			200	0.6	0.5	3.5	0.4

Figure S7. Antigen formulations for printing the *S. aureus* antigens into the array on the Si/SiO_2 substrate used for measurements reported in figures S6 and 7. Avidin and biotin are not required for LTR to return accurate thickness measurements. These were substrates originally prepared for AIR where the avidin/biotin system was used as a failed attempt to boost the probe thickness.

Protein Binding Model for LTR:

As discussed in the text, the bound protein on the LTR probe surface can be described using the one-site Langmuir binding isotherm model [25,17] by the equation

$$\Gamma = \frac{C}{C + K_D}, \qquad (S1)$$

where Γ is the fraction of available binding sites filled, C is the concentration of the antigen of interest in the measured solution (typically human serum), and K_D is the dissociation constant of the capture antibody attached to the probe surface. The measured thickness change h for the LTR probe for a particular analyte concentration is then given by

$$h = \Gamma\, h_{max}, \qquad (S2)$$

where h_{max} is the maximum thickness change when all available binding sites on the probe are filled. h_{max} is measured experimentally by creating a calibration curve, but can also be estimated geometrically from the antigen and antibody sizes for a particular analyte (or for other probe/target binding pairs; here we focus on antibody/antigen).

An effective thickness change averaged over a region much larger than the antigen size (as in the case of LTR, or any optical thin-film measurement with analytes much smaller than the wavelength of light used) for a single bound antigen is equal to the volume of the antigen divided by the area of the measured region. The maximum thickness change is equal to the thickness change per particle multiplied by the maximum number of bound antigen particles:

$$h_{max} = \frac{N_{max} V_{antigen}}{A_{total}} \qquad (S3)$$

The maximum number of antigen particles is equal to the number of antibodies bound to the surface, multiplied by the number of available binding sites per antibody γ (even for molecules with multiple binding sites, the one-site Langmuir model can be used as long as the binding affinity for each site is the same) and the fraction of available binding sites f_α (since, in practice, due to random antibody orientation on the surface and steric hindrance, especially if the antigen size is comparable to that of the antibody, not all possible binding sites will be available to bind):

$$N_{max} = \gamma\, f_\alpha\, N_{antibody} \qquad (S4)$$

If they are unknown, the volumes of the antibody and antigen proteins can be estimated from their molecular weights using the equation [*]

$$V_{protein}(nm^3) \approx 1.212 \times M(kDa). \qquad (S5)$$

The average number of antibodies that can bind in a monolayer on an area A_{total} much larger than the antibody size is equal to that area divided by the two-dimensional area occupied by each antibody $A_{antibody}$ and multiplied by the areal fill fraction of the antibodies f_f:

$$N_{antibody} = f_f \frac{A_{total}}{A_{antibody}}. \qquad (S6)$$

Often the average area occupied by an antibody is not well characterized. However, a minimum average area can be calculated by assuming the protein volume is spherical [*], giving a radius of $r_{min} = \sqrt[3]{\frac{3V_{antibody}}{4\pi}}$ and an area of $A_{min} = \pi r_{min}^2 = \sqrt[3]{\pi\left(\frac{3V_{antibody}}{4}\right)^2}$. For closely packed spheres in a triangular/hexagonal lattice, the maximal two-dimensional fill fraction is equal to $f_f = \frac{\pi}{2\sqrt{3}} \approx 0.907$ (although this decreases to $f_f \approx 0.886$ for two-dimensional arrangements of spheres packed randomly [**]).

It should be acknowledged that in reality the volume of the protein is *not* organized into a simple sphere – thus, the average area occupied by each protein will be larger than A_{min}. Additionally, the packing density may be significantly lower than the spherical packing limit of ~ 0.886. For the purposes of this calculation, both these effects can be accounted for by using a lower, effective fill fraction term. Of course, a high fill fraction does not always translate to more binding experimentally, since close packing of antibodies may interfere with antigen binding (decrease f_α). Since in practice the binding curve for a particular antibody/antigen pair will be measured experimentally, a reasonable estimate for the effective f_f can be chosen to estimate h_{max} with appropriate error bounds.

Using this fact, along with equations S4 and S6, equation S3 can be rewritten as

$$h_{max} = \frac{\gamma f_f f_\alpha V_{antigen}}{A_{antibody}} \approx \frac{\gamma f_f f_\alpha V_{antigen}}{\pi r_{antibody}^2} = \frac{\gamma f_f f_\alpha V_{antigen}}{\sqrt[3]{\pi\left(\frac{3V_{antibody}}{4}\right)^2}} \qquad (S7)$$

as a theoretical estimate that can be used to predict the maximum possible binding for a given antibody/antigen pair, and (in conjunction with equation S1) the binding that could be observed for a given antigen concentration.

In the case of LTR, a primary antigen of interest is IL-6 ($M \approx 26\ kDa$) with a corresponding monoclonal antibody ($M \approx 150\ kDa, K_D \approx 1\ nM, \gamma = 2$ binding sites). The fill fraction was estimated to be $f_f = 0.45 \pm 0.2$ from an ellipsometry study which found an adsorption density of $3\,\frac{mg}{m^2}$ of comparable IgG antibodies onto a silica substrate[***], with the relatively large error bounds included to account for the differences between the methods used in that study and in LTR experiments. (With their molecular weight of 150 kDa, equation S5 gives a spherical area of $A_{sph} = 38.8\ nm^2$, while the adsorption density indicates that the actual average area per antibody – the inverse of the particle density – is $A = \frac{1}{n[nm^{-2}]} = \frac{(1.5\times10^5\ [g/mol])\ (10^9\ nm/m)^2}{(3\times10^{-3}\ [g/m^2])\ (6.022\times10^{23}[mol^{-1}])} = 83.0\ nm^2$. This results in an effective fill fraction of $f_f = \frac{A_{sph}}{A} = 0.467$.) The fraction of available binding sites was estimated to vary between 0.5 and 1 (0.75 ± 0.25) since the antibodies are deposited randomly on the substrate and the resulting quasi-random orientation is not well characterized. Using equation S7 and independent propagation of uncertainties, these values give an estimate of $h_{max} = 5.5 \pm 3.0$ Å. As discussed in the text, the measured value of h_{max} was 6 Ångstroms, in good agreement with this estimate.

[*] Erickson, H.P. Size and Shape of Protein Molecules at the Nanometer Level Determined by Sedimentation, Gel Filtration, and Electron Microscopy. *Biol Proced Online* 2009, 11:32. DOI: 10.1007/s12575-009-9008-x

[**] Zaccone, A. Explicit Analytical Solution for Random Close Packing in d=2 and d=3. *Phys. Rev. Lett.* **2022**, 128, 028002. DOI:10.1103/PhysRevLett.128.028002

[***] Malmsten, M. Ellipsometry Studies of Protein Layers Adsorbed at Hydrophobic Surfaces. *J. Colloid Interface Sci.* **1994**,166: 2 (1994). DOI:10.1006/jcis.1994.1303.
